# Relationship Between Apolipoprotein E Genotypes, Unhealthy Weight Status, and Cognitive Impairment in Older Adults of Predominantly African Descent

**DOI:** 10.3390/diseases13120394

**Published:** 2025-12-06

**Authors:** Jean-Pierre Clotilde, Livy Nicolas, Laurent Larifla, Fritz-Line Velayoudom, Stanie Gaete, Yann Ancedy, Ingrid Cirederf, Rosan Fanhan, Lydia Foucan

**Affiliations:** 1 Medical Unit, Médical Centre Lucien NI COLAS, Clinique Nouvelles Eaux Marines, 97160 Le Moule, Guadeloupe, France; jp.clotilde@clinique-eauxmarines.com (J.-P.C.); l.nicolas@clinique-eauxmarines.com (L.N.); cirederf_ingrid@hotmail.fr (I.C.); rosan.fanhan@wanadoo.fr (R.F.); 2 Research Team on Cardiometabolic Risk ECM, University Hospital, University of the Antilles, 97157 Pointe-à-Pitre, Guadeloupe, France; laurent.larifla@chu-guadeloupe.fr (L.L.); fritz-line.velayoudom@univ-antilles.fr (F.-L.V.); 3 Cardiology Unit, University Hospital, University of the Antilles, 97157 Pointe-à-Pitre, Guadeloupe, France; 4 Karubiotec, University Hospital, 97157 Pointe-à-Pitre, Guadeloupe, France; stanie.gaete@chu-guadeloupe.fr; 5 Clinical Research Unit, Médical Centre Lucien NICOLAS, Clinique Nouvelles Eaux Marines, 97160 Le Moule, Guadeloupe, France; yann.ancedy@gmail.com

**Keywords:** *APOE* gene, aging, cognitive impairment, frailty, unhealthy weight

## Abstract

**Background:** Apolipoprotein E4 (*APOE4*) represents a major genetic risk factor for Alzheimer’s disease. **Objectives:** We aimed to analyze the relationship between cognitive impairment (CI), unhealthy weight status, and *APOE* genotypes in individuals of predominantly African descent aged 55 years and more. Genotyping of two single-nucleotide polymorphisms, rs7412 and rs429358, of the *APOE* gene was performed. **Results**: Among 310 individuals, the mean age was 75.64 years, the mean BMI was 25.94 kg/m^2^, and the prevalence of CI was 18.1%. Most subjects were ε3/ε3 carriers (49%), while ε2-carriers and ε4-carriers represented 14.5% and 36.5%, respectively. Older age, the presence of undernutrition, and *APOE4* carriers were more frequently found in underweight vs. non-underweight individuals and in those with CI vs. those without CI. The adjusted odds ratios for prevalent CI were nearly four times higher for underweight individuals compared to obese individuals. Those carrying two ε4 alleles exhibited three times the odds of CI (OR = 3.31 (95% CI: 1.15–9.91), *p* = 0.026) compared to those with no ε4 alleles. **Conclusions:** In this cross-sectional study, being underweight and carrying the *ApoE* ε4 allele were independently associated with cognitive impairment. These findings suggest that monitoring weight changes and *APOE* genotypes in older adults may have clinical significance.

## 1. Introduction

Cognitive impairment (CI), a progressive neurocognitive disorder, is characterized as decline in brain functions such as memory, concentration, and problem-solving. This disorder can be found in those who are underweight or obese, two forms of unhealthy weight which are common in older people and are both associated with morbidity.

In fact, some consequences of underweight and undernutrition, especially in the elderly, include impaired muscle function, decreased immune function, frequent illnesses [1], and reduced cognitive function [1,2,3]. A loss of lean mass in older adults has been linked with brain atrophy and cognitive performance [4], and sarcopenia has been associated with parietal gray matter volume atrophy in a middle-aged population [5].

In parallel, obesity is linked to inflammation, which is responsible for the pathway leading to atherosclerosis [6], which is itself associated with a faster decline in global cognition [7,8].

Regarding cognitive impairment (CI), its relationship with overweight/obesity may be different depending on the stage of life and is therefore a source of discussion. The results of a review and meta-analysis of longitudinal studies highlight not only a positive association between obesity in mid-life and dementia in old age but also a negative association in later life [9].

The apolipoprotein E (*APOE*) gene is known to impact the onset of neurodegenerative diseases [10]. This gene, containing four exons and three introns, is mapped on the long arm of chromosome 19 (19q13.2) [11]. Genetic variation at the *APOE* locus induces three frequent isoforms, *APOE2* (Cys112, Cys158), *APOE3* (Cys112, Arg158), and *APOE4* (Arg112, Arg158), which are encoded by the ε2, ε3, and ε4 alleles, respectively [12,13]. The association of *APOE4* with an increased risk of atherosclerosis, impaired cognitive function, and Alzheimer’s disease (AD) has been well-documented [11,14]. Mild cognitive impairment (MCI) has been recognized as the preclinical and transitional stage between healthy aging and dementia [15]; thus, more research is needed to better understand the association between various findings observed in CI in order to implement effective interventions and delay progression to dementia.

The population of Guadeloupe has been estimated at approximately 472,124 individuals. It is composed of 80% subjects of African descent (a predominantly mixed population), approximately 8% descendants of Indian migrants, mainly from southern India, and 12% subjects from other ethnic groups.

To our knowledge, in this population, the relationship between *APOE* genotypes and cognitive disorders has never been analyzed, and their impact on the relationship between unhealthy weight and CI needs to be investigated.

The objective of this study was to analyze the relationship between cognitive impairment (CI), unhealthy weight, and *APOE* genotype status in individuals of predominantly African descent.

## 2. Patients and Methods

In this study, we considered subjects of both sexes, aged 55 years and older, from the SMet-Age study conducted between 2021 and 2023 on the island of Guadeloupe. SMet-Age is a cross-sectional study on metabolic syndrome in patients who were admitted to the medical unit in a health establishment in Guadeloupe.

Patients with Alzheimer’s disease (AD) or dementia were excluded and participants with a cancer diagnosis were not excluded.

The study was registered at the French National Agency for Medicine and Health Products Safety Security (Internal Reference ANSM-RCB-PAR 1602697118, National N° 2020-A02851-38).

### 2.1. Data Collection

For each patient, we collected the following data: age, personal medical history, treatment at entry, use of antihypertensive or antidiabetic treatments, and biological data at entry.

Height and weight were measured and body mass index (BMI) was defined as weight (kg) divided by the square of height (m).

Blood pressure was measured according to a standardized protocol with automatic sphygmomanometers.

The measurements were conducted by trained nurses and physicians.

The atherogenic index of plasma (AIP) was calculated as the logarithmic transformation of the ratio of triglycerides to high-density lipoprotein cholesterol (log [TG/HDL-C]).

In the present study, cognitive function was measured using the Abbreviated Mental Test Score (AMTS), a 10-point assessment that was introduced by Hodkinson in 1972 to rapidly assess elderly patients for the possibility of dementia [16].

### 2.2. Genotyping of APOE

Genotyping of the two single-nucleotide polymorphisms (SNPs), rs7412 (NC_000019.10: g.44908822 C > T; Arg158Cys) and rs429358 (NC_000019.10: g.44908684 T > C, Cys112Arg), of the *APOE* gene was performed using the Taqman^®^ technique following the manufacturer’s guidelines (Life Technologies ref. 437135).

These SNPs contain alleles of the major *ApoE* isoforms: ε2, ε3, and ε4. Only SNPs with a genotyping call rate (QV) greater than 95% were used in the analyses.

*APOE* genotype status was defined by *APOE2* (ε2/ε2 or ε2/ε3), *APOE3* (ε3/ε3), and *APOE4* (ε3/ε4 or ε4/ε4). Carriers of ε2/ε4 were not considered given the fact that these alleles have opposite effects on cognitive decline [17]. The number of *ApoE* ε4 alleles was also considered.

### 2.3. Clinical Parameters

*Cognitive impairment*: Individuals with CI had an AMTS < 6 and experienced persistent memory loss or symptoms of confusion, noted in the medical report, despite this not being assessed by the AMTS [16].

*Weight status*: Since the number of individuals with a BMI < 18.5 kg/m^2^ was very small, we used the threshold of 21 kg/m^2^ for identification of underweight. Subjects with a BMI < 21 kg/m^2^ were classified as underweight (G1), those with a BMI of 21–24.9 kg/m^2^ were considered normal weight (G2), those with a BMI 25–29.9 kg/m^2^ were classified as overweight (G3), and those with a BMI ≥ 30 kg/m^2^ were classified as obese (G4).

*Hypertension*: Systolic blood pressure (SBP) ≥ 140, diastolic blood pressure (DBP) ≥ 90 mmHg, or history of hypertension and current use of antihypertensive medication were used to classify patients as having hypertension.

*Diabetes–high fasting blood glucose* (FBG): This was defined as known diabetes mellitus or FBG ≥ 5.6 mmol/L (100 mg/dL).

*Frailty* was identified based on a Study of Osteoporotic Fractures (SOF) Index ≥ 2 [18].

*Dependence* was identified based on a Lawson Instrumental Activities of Daily Living (IADL) score ≥ 2 [19].

We considered a prealbumin level < 0.20 g/L or an albumin level < 30 g/L as *biomarkers of undernutrition*.

### 2.4. Statistical Analysis

The characteristics of the sample are presented as means with standard deviations or the median value and range for continuous variables and numbers (percentages) for categorical variables.

The chi-square test was used to test percentage differences between groups. To test mean differences, we used analysis of variance (ANOVA).

Baseline characteristics were compared across BMI categories and according to the presence/absence of CI and to *APOE* genotype status.

We used multivariable logistic regression models adjusted for sex, age, weight status, *APOE4* genotype status (model 1), and the number of ε4 alleles (model 2) to analyze the effects of underweight on the risk of CI.

Adjusted odds ratios (ORs) and 95% confidence intervals (95% CIs) were estimated.

IBM SPSS Statistics software version 21 was used for data analyses. All tests were two-sided and a *p* value < 0.05 was considered significant.

## 3. Results

Overall, 310 subjects were included.

The characteristics of the study sample are presented in Table 1.

The mean age was 75.6 ± 9.5 years (range 56 to 98 years), the median AIP value was −0.0984 (range −1.60 to 1.88), and women predominated at 56.1%. Among the subjects, 19.4% were underweight, 20.6% were obese, 40.4% had diabetes, and 18.1% exhibited CI.

Among the study sample, no participant carried ε2/ε4. Most subjects were ε3/ε3 carriers (n = 152; 49%), 113 subjects (36.5%) were ε4 carriers (carrying at least one ε4 allele), and 45 (14.5%) were ε2 carriers (carrying at least one ε2 allele).

Considering BMI categories, CI (*p* = 0.003), frailty (*p* < 0.001), dependance (*p* < 0.001), a prealbumin level < 0.20 g/L (*p* = 0.025), an albumin level < 30 g/L (*p* = 0.003), and *APOE4* carriers (*p* = 0.038) were more frequently found in the underweight group than in the other BMI groups, as shown in Table 1.

An elevated AIP (AIP > than the median value) was more frequently found in overweight and obese individuals (*p* = *0.009*).

Compared with individuals without CI, those with CI more frequently had an age ≥ 75 years (*p* = 0.007), were underweight (*p* < 0.001), and had frailty (*p* = 0.029), dependance (*p* < 0.001), and a prealbumin level < 0.20 g/L (*p* = 0.012).

The number of *APOE4* genotype carriers and *ApoE* ε4/ε4 allele carriers was also higher among individuals with CI compared with those without CI ((48.2% vs. 33.9%; *p* = 0.043) and (12.5% vs. 5.1% *p* =0.042), respectively).

Regarding *APOE* genotypes status, the prevalence of CI was 11.1%, 15.8%, and 23.9%; *p* = 0.100 in *APOE2*, *APOE3*, and *APOE4* carriers, respectively.

However, this prevalence was significantly higher in *APOE4* carriers than in non-carriers (23.9% vs. 14.7%; *p* = 0.001). Underweight individuals more frequently carried *APOE4* (*p* = 0.025).

*ApoE* ε4/ε4 carriers had a higher frequency of CI (35.0%) compared with those with one ε4 allele (21.5%) and those without an ε4 allele (14.1%) *(p* = 0.047), as shown in Table 2. The difference in CI frequency between ε3/ε4 carriers and ε4/ε4 carriers was not significant (*p* = 0.159).

The odds ratios for experiencing CI in the 310 individuals with complete data for the co-variables are presented in Table 3.

Compared with obese individuals, underweight individuals exhibited nearly four times the odds of CI in both models. Older age (age > 75 years) remained significantly associated with CI in both models.

Individuals with one ε4 allele had non-significant odds of prevalent CI compared to those with no ε4 allele (OR = 1.50 (95% CI: 0.77–2.93), *p* = 0.230). Those carrying two ε4 alleles exhibited three times the odds of CI (OR = 3.31 (95% CI: 1.15–9.91), *p* = 0.026) compared to those with no ε4 alleles.

Considering the ε4 carriers alone (N =113) and compared with ε3/ε4 carriers, the OR of prevalent CI was 2.09 ((95% CI 0.70–6.27); *p* = 0.188) for the ε4/ε4 carriers.

## 4. Discussion

This study has explored the relationship between unhealthy weight and CI and considered, for the first time in this Caribbean population, the joint effects of *APOE* genotypes in individuals mainly of African descent.

We observed that underweight was positively and significantly associated with CI independently of *APOE* status. Additionally, the *ApoE* ε4 allele was associated with prevalent CI in this population.

In these individuals aged 55 years and older, we found a prevalence of 18.1% for CI, which is not very far from the prevalence of 15.56% for MCI among community-dwelling adults aged 50 years and older that has been reported in a meta-analysis and systematic review [20].

### 4.1. Unhealthy Weight, Frailty, Undernutrition, Atherosclerosis, and Cognitive Impairment

Frailty was more frequently found in underweight individuals (96.9%) and in those with CI (80.4%). To identify frailty, we used the SOF index, which has been validated in [18]. This syndrome includes some physical markers such as a lower BMI, loss of grip strength, and loss of physiologic reserve, which lead to increased vulnerability and adverse health outcomes including cognitive decline [21,22,23]. Frailty and cognitive impairment are often present concomitantly, and their simultaneous presence, in the absence of dementia, constitutes cognitive frailty syndrome [22].

In this study, underweight individuals and those with CI were also older and had higher prevalence of low pre-albumin and low albumin levels, both of which are markers of undernutrition.

These results suggest that underweight induced by undernutrition could be one explanation of CI in the elderly.

Our results in these older adults also show that underweight individuals had higher odds of prevalent CI compared to obese individuals. Although 45.3% of our obese individuals also exhibited frailty, other explanations are put forward to account for the prevalence of CI. Obesity, considered a complex disease, is often associated with various comorbidities, including type 2 diabetes mellitus (T2DM), cardiovascular diseases (CVDs) and others [24]. A high prevalence of cognitive decline has been reported in diabetic populations [25], and longitudinal studies have shown accelerated decline in cognitive functions in patients with T2DM [26]. Obesity is also frequently associated with other vascular risk factors, such as hypertension and dyslipidemia, which induce atherosclerosis. An elevated atherogenic index of plasma (AIP), calculated from log [TG/HDL-C], has been found to be associated with CI in adult Americans [27]. Moreover, in a review of observational and postmortem studies, intracranial atherosclerosis disease, which alters cerebrovascular hemodynamics and the structural integrity of the brain, was associated with CI and dementia across age, sex, and race groups [8]. The mean value of the AIP, which was significantly higher in our obese individuals, was not significantly different between our individuals with and without CI.

### 4.2. ApoE Gene and Cognitive Impairment

Cognitive decline precedes and predicts functional decline in aging and Alzheimer’s disease (AD) [28], a progressive neurodegenerative disease for which the *APOE* gene is among its greatest risk [17].

The *APOE* gene encodes apolipoprotein E, a lipoprotein involved in the cerebral transport and metabolism of lipids, which are necessary for neuronal maintenance [29,30].

The three major isoforms of the *APOE* gene, *APOE2*, *APOE3*, and *APOE4*, differ from each other only by single-amino-acid substitutions, but these changes have profound functional consequences.

We noted a non-significant lower *APOE2* genotype frequency in our individuals with CI compared to those without CI.

Using data of patients with subcortical vascular cognitive impairment, in a three-year longitudinal study and compared to the *APOE3* homozygotes, *APOE2* had protective effects against amyloid-ß accumulation, cortical thinning, and cognitive decline [31].

Carrying ε2 or ε3 alleles appeared to be protective against AD in various studies [29,31,32,33,34].

Conversely, in our study, *ApoE* ε4 was positively associated with CI, and the individuals carrying ApoE ε4/ε4 had an odds ratio of prevalent CI 3.31 times greater compared to those with no ε4 alleles.

It has been reported that SCD individuals carrying *ApoE* ε4 showed worse cognitive decline [35] and more severe brain structural damage [36] than those with SCD or *ApoE* ε4 alone.

Moreover, 85% of elderly *ApoE* ε4/ε4 individuals (average age, 81) scored in the unimpaired range on a screening mental status test in a previous study [10].

Concerning the association of *ApoE* ε4 with the risk of AD, a meta-analysis of clinical- and autopsy-based studies demonstrated that, compared with individuals with an ε3/ε3 genotype, the risk of AD was increased in carriers of *ApoE* ε4/ε4 (OR 14.9) among Caucasian subjects [33], but this association was weaker among African Americans [33].

A non-significant higher frequency of *APOE2* carriers was noted in our obese older adults, whereas a significantly higher frequency of *APOE4* carriers was noted in those with underweight compared with the other groups. Women with at least one *ApoE* ε4 allele have been reported to experience a more rapid decline in BMI after reaching 70 years of age compared to women without this allele [37].

In parallel, obesity has been associated with cognitive decline in early-elderly cognitively normal individuals, in *APOE2* carriers and in *APOE3* homozygote individuals, but not in *APOE4* carriers [38]. But, for these authors, obesity may accelerate cognitive decline in middle- to early-elderly cognitively normal individuals without *APOE4*, likely by provoking vascular impairments [38].

### 4.3. Relationship Between Lower BMI, Cortical Amyloid Burden, and APOE Gene

Several studies support the relationship between BMI and cortical amyloid burden. Biomarker levels reflective of AD pathophysiology, including beta amyloid and tau, were associated with a lower BMI in a sample of cognitively normal MCI and AD participants [39]. It has been found that lower baseline late-life BMI was associated with greater PiB retention, an established biomarker for cortical amyloid deposition and increased risk for future development of AD dementia [23]. Additionally, unintentional weight loss is often observed in the preclinical stages of dementia [40], and long-term body weight variability has been found to be significantly associated with a high risk of dementia [41].

Alzheimer’s disease is characterized primarily by a loss of cognitive function, and *APOE* 4 represents a major genetic risk factor for AD. This increased risk has been linked to increased cortical Aβ accumulation [42,43].

In a recent study of the National Alzheimer’s Coordinating Center (NACC) examining the prevalence of CI in subjects carrying *APOE4* alleles [44], across all ethnic groups, except for Pacific Islanders, an increase in *APOE4* count was associated with a higher prevalence and an earlier onset of CI [44]. In this study, compared to individuals with no ε4 allele, those with two ε4 alleles exhibited nearly four times the odds of CI (OR = 3.75, *p* < 0.001). We found very similar results for our older adults of predominantly African descent with higher odds of CI (OR = 3.31, *p* = 0.026) in carriers of two ε4 alleles.

Not all our patients who are *APOE4* carriers, even those who are *ApoE* ε4/ε4 carriers, and not all our underweight patients show findings of CI, recalling that there are other risk factors for cognitive decline including age, cardiovascular disease risk factors, education level, and social engagement. Moreover, complex gene–gene interactions between *APOE* and other genetic risk factors for cognition have been reported [17]. There may be a vicious cycle in older subjects including a loss of appetite followed by undernutrition, weight loss, and frailty syndrome that leads to cognitive decline, which can lead to forgetting to eat and becoming underweight. Carrying *APOE4* could impact the onset of cognitive disorder but the effective role of this genotype is not completely understood. These findings reveal phenotypic associations involving body weight, genotypes, and cognition but the underlying pathophysiological mechanisms require confirmation through biomarker studies.

### 4.4. Limitations

The limitations of our study include the small sample size that may not be representative of the population. Additionally, the cross-sectional design does not allow us to determine if underweight is a cause or a consequence of CI and how the *ApoE* ε4 allele mediates this relationship.

Additionally, we did not take into account confounders such as education level, socio-economic status, and mental health history of patients. Possible selection bias may be related to the results of the AMTS since this test is not a substitute for a full cognitive assessment.

Despite these limitations, this is the first study exploring the relationship between BMI, CI, and *APOE* genotypes in our population of predominantly African descent, and which provides information that could contribute to improving patient care.

## 5. Conclusions

In this cross-sectional study, underweight and carriage of the *ApoE* ε4 allele were independently associated with cognitive impairment. These findings suggest that monitoring weight changes and *APOE* genotype status in older adults may have clinical significance; however, the causal relationship between these factors and the underlying mechanisms require clarification through larger longitudinal studies.

## Figures and Tables

**Table 1:** Characteristics of patients according to weight status and presence/absence of cognitive impairment.

			Weight Status					Cognitive Impairment		
	N	Overall 310	G1 60	G2 90	G3 96	G4 64	*p*	No 254	Yes 56	*p*
Age > 75 years (%)	310	**57.1**	66.7	57.8	59.4	43.8	0.068	53.5	75.2	**0.007**
Gender (men) (%)	310	**43.9**	50.0	43.3	46.9	34.4	0.305	42.5	50	0.307
Diabetes–High FBG (%)	307	**40.4**	35.0	33.0	47.4	45.3	0.150	39.3	45.5	0.398
Weight status	310									
Obesity		**20.6**	**---**	**---**	**---**	**---**	**---**	22.8	10.7	**0.043**
Overweight		**31.0**	**---**	**---**	**---**	**---**	**---**	32.7	23.2	0.166
Normal weight		**38.1**	**---**	**---**	**---**	**---**	**---**	28.7	30.4	0.809
Underweight		**19.4**	**---**	**---**	**---**	**---**	**---**	15.7	35.7	**0.001**
Hypertension	296	**51.4**	35.1	47.7	57.1	62.9	**0.012**	53.1	43.4	0.201
Cognitive impairment (%)	310	**18.1**	33.3	18.9	13.5	9.4	**0.003**	**---**	**---**	**---**
Frailty (%)	310	**68.1**	95.0	77.8	57.3	45.3	**<0.001**	65.4	80.4	**0.029**
Dependance (%)	310	**18.1**	38.3	13.3	15.6	9.4	**<0.001**	10.2	53.6	**<0.001**
Prealbumin level < 0.20 g/L (%)	304	**39.5**	53.3	42.7	35.1	27.9	**0.024**	36.1	54.5	**0.012**
Albumin level < 30 g/L (%)	308	**11.0**	31.3	11.9	4.2	9.5	**0.003**	11.1	10.7	0.932
AIP > −0.098	299	49.8	39.0	40.2	59.1	60.0	**0.009**	51.2	43.4	0.302
*APOE2* carriers (%)	310	**14.5**	6.7	14.4	15.6	20.3	0.186	15.7	8.9	0.190
*APOE3* carriers (%)	310	**49.0**	43.3	48.9	57.3	42.2	0.203	50.4	42.9	0.307
*APOE4* carriers (%)	310	**36.5**	50.0	36.7	27.1	37.5	**0.038**	33.9	48.2	**0.043**
*apoE* ε4/ε4 carriers (%)	310	**6.5**	9.4	6.8	6.3	4.7	0.847	5.1	12.5	**0.042**

Data are presented as column percentages for qualitative variables. G1: underweight—BMI < 21 kg/m^2^; G2: normal weight—BMI 21–24.9 kg/m^2^; G3: overweight—BMI 25–29.9 kg/m^2^; G4: obesity—BMI ≥ 30 kg/m^2^. *APOE* genotype status: *APOE2* (ε2/ε2 or ε2/ε3), *APOE3* (ε3/ε3), and *APOE4* (ε3/ε4 or ε4/ε4). AIP: atherogenic index of plasma calculated as logarithmic transformation of ratio of triglycerides to high-density lipoprotein cholesterol, and −0.098 is AIP median value. Frailty: Study of Osteoporotic Fractures (SOF) Index ≥ 2. Dependance: Lawson Instrumental Activities of Daily Living (IADL) score ≥ 2. Significant *p*-values are in bold.

**Table 2:** Characteristics of patients according to *APOE* genotype status and number of *ApoE* ε4 alleles.

			*APOE* Genotypes				Number of *ApoE* ε4 Alleles			
N		ALL 310	*APOE* 2 45	*APOE* 3 152	*APOE* 4 113	*p*	0 297	1 93	2 20	*p*
Age > 75 years (%)	310	57.1	57.8	60.5	52.2	0.399	59.9	52.7	50.0	0.411
Gender (men) (%)	310	43.9	42.2	46.1	41.6	0.748	45.2	41.9	40.0	0.819
Diabetes–High FBG (%)	307	40.4	40.0	39.7	41.4	0.960	39.8	43.0	33.3	0.717
AIP > −0.098	299	49.8	47.6	56.1	42.2	0.085	54.2	41.1	47.4	0.120
Obesity (%)	310	20.6	28.9	17.8	21.2	0.264	20.3	22.6	15.0	0.735
Underweight (%)	310	19.4	8.9	17.1	26.5	**0.025**	15.2	26.9	25.0	0.051
Cognitive impairment (%)	310	18.1	11.1	15.8	23.9	0.100	14.7	21.5	35.0	**0.047**

Data are presented as column percentages for qualitative variables. Diabetes–High FBG: known diabetes or FBG ≥ 5.6 mmol/L (100 mg/dL). *APOE* genotype status: *APOE2* (ε2/ε2 or ε2/ε3), *APOE3* (ε3/ε3), and *APOE4* (ε3/ε4 or ε4/ε4). AIP: atherogenic index of plasma calculated as logarithmic transformation of ratio of triglycerides to high-density lipoprotein cholesterol; −0.098 is AIP median value. Significant *p*-values are in bold.

**Table 3:** Multivariate logistic regression of the association between cognitive impairment, weight status, *APOE4* genotype, and *ApoE* ε4 alleles in 310 hospitalized adults aged 55 years and older.

	Model 1 *			Model 2 **		
	OR	95% CI	*p*	OR	95% CI	*p*
				1		
Age ≥ 75 years	2.29	(1.18–4.48)	**0.015**	2.33	(1.19–4.54)	**0.013**
Gender (men)	1.32	(0.72–2.43)	0.368	1.34	(0.73–2.47)	0.345
Weight status						
Obesity	1	----	----	1		
Overweight	1.35	(0.48–3.84)	0.572	1.31	(0.46–3.74)	0.613
Normal weight	1.94	(0.71–5.34)	0.199	1.91	(0.70–5.28)	0.210
Underweight	3.67	(1.32–10.2)	**0.013**	3.66	(1.31–10.2)	**0.013**
*APOE4* genotype	1.76	(0.95–3.27)	0.072	----	----	----
Number of ε4 alleles						
No ε4	----	----	----	1		
One ε4	----	----	----	1.60	(0.77–2.93)	0.230
Two ε4	----	----	----	3.31	(1.15–9.91)	**0.026**

*APOE4*: (ε3/ε4 or ε4/ε4). Both models were adjusted for age, gender, weight status (with obesity as the reference group), *APOE4* status (in model 1 *), and *ApoE* ε4 alleles (in model 2 **), with no ε4 as the reference. OR (95% CI): odds ratio (95% confidence interval). Significant *p*-values are in bold.

## Data Availability

The data presented in this study are available upon reasonable request from the corresponding author. The data are not publicly available because the data are part of ongoing research.

## Abbreviations

Aβ	Amyloid-β
AD	Alzheimer’s disease
AIP	Atherogenic index of plasma
AMTS	Abbreviated mental test score
APOE	Apolipoprotein E
BMI	Body mass index
MCI	Cognitive impairment
FBG	Fasting blood glucose
ADL	Instrumental activities of daily living
MCI	Mild cognitive impairment
MRI	Magnetic resonance imaging
OR	Odds ratio
SCD	Subjective cognitive decline
SOF	Study of osteoporotic fractures
SNP	Single nucleotide polymorphisms
T2DM	Type 2 diabetes mellitus

## References

[1] Chapman I.M. (2006). Nutritional disorders in the elderly. Med. Clin. N. Am..

[2] Nicolas L., Bassien-Capsa V., Ancedy Y., Chingan-Martino V., Clotilde J.P., Afassinou Y.M., Galantine O., Fanhan R., Tabue-Teguo M., Foucan L. (2024). Associations between Cognitive Impairment, Weight Status and Comorbid Conditions in Hospitalized Adults of 55 Years and Older in Guadeloupe. Healthcare.

[3] Dong W., Kan L., Zhang X., Li M., Wang M., Cao Y. (2023). Association between body mass index and cognitive impairment in Chinese older adults. Front. Public Health.

[4] Burns J.M., Johnson D.K., Watts A., Swerdlow R.H., Brooks W.M. (2010). Reduced lean mass in early Alzheimer disease and its association with brain atrophy. Arch. Neurol..

[5] Yu J.H., Kim R.E.Y., Jung J.M., Park S.Y., Lee D.Y., Cho H.J., Kim N.H., Yoo H.J., Seo J.A., Kim S.G. (2021). Sarcopenia is associated with decreased gray matter volume in the parietal lobe: A longitudinal cohort study. BMC Geriatr..

[6] Csige I., Ujvarosy D., Szabo Z., Lorincz I., Paragh G., Harangi M., Somodi S. (2018). The Impact of Obesity on the Cardiovascular System. J. Diabetes Res..

[7] Liang J., Pan Y., Zhang W., Gao D., Ma J., Zhang Y., Ji M., Dai Y., Liu Y., Wang Y. (2024). Associations Between Atherosclerosis and Subsequent Cognitive Decline: A Prospective Cohort Study. J. Am. Heart Assoc..

[8] Sabayan B., Goudarzi R., Ji Y., Borhani-Haghighi A., Olson-Bullis B.A., Murray A.M., Sedaghat S. (2023). Intracranial Atherosclerosis Disease Associated with Cognitive Impairment and Dementia: Systematic Review and Meta-Analysis. J. Am. Heart Assoc..

[9] Li J., Joshi P., Ang T.F.A., Liu C., Auerbach S., Devine S., Au R. (2021). Mid- to Late-Life Body Mass Index and Dementia Risk: 38 Years of Follow-up of the Framingham Study. Am. J. Epidemiol..

[10] Hyman B.T., Gomez-Isla T., Briggs M., Chung H., Nichols S., Kohout F., Wallace R. (1996). Apolipoprotein E and cognitive change in an elderly population. Ann. Neurol..

[11] Mahley R.W., Rall S.C. (2000). Apolipoprotein E: Far more than a lipid transport protein. Annu. Rev. Genom. Hum. Genet..

[12] Giau V.V., Bagyinszky E., An S.S., Kim S.Y. (2015). Role of apolipoprotein E in neurodegenerative diseases. Neuropsychiatr. Dis. Treat..

[13] Singh P.P., Singh M., Mastana S.S. (2006). APOE distribution in world populations with new data from India and the UK. Ann. Hum. Biol..

[14] Genin E., Hannequin D., Wallon D., Sleegers K., Hiltunen M., Combarros O., Bullido M.J., Engelborghs S., De Deyn P., Berr C. (2011). APOE and Alzheimer disease: A major gene with semi-dominant inheritance. Mol. Psychiatry.

[15] Anderson N.D. (2019). State of the science on mild cognitive impairment (MCI). CNS Spectr..

[16] Hodkinson H.M. (1972). Evaluation of a mental test score for assessment of mental impairment in the elderly. Age Ageing.

[17] Fan J., Tao W., Li X., Li H., Zhang J., Wei D., Chen Y., Zhang Z. (2019). The Contribution of Genetic Factors to Cognitive Impairment and Dementia: Apolipoprotein E Gene, Gene Interactions, and Polygenic Risk. Int. J. Mol. Sci..

[18] Kiely D.K., Cupples L.A., Lipsitz L.A. (2009). Validation and comparison of two frailty indexes: The MOBILIZE Boston Study. J. Am. Geriatr. Soc..

[19] Lawton M.P., Brody E.M. (1969). Assessment of older people: Self-maintaining and instrumental activities of daily living. Gerontologist.

[20] Bai W., Chen P., Cai H., Zhang Q., Su Z., Cheung T., Jackson T., Sha S., Xiang Y.T. (2022). Worldwide prevalence of mild cognitive impairment among community dwellers aged 50 years and older: A meta-analysis and systematic review of epidemiology studies. Age Ageing.

[21] Buchman A.S., Schneider J.A., Leurgans S., Bennett D.A. (2008). Physical frailty in older persons is associated with Alzheimer disease pathology. Neurology.

[22] Kelaiditi E., Cesari M., Canevelli M., van Kan G.A., Ousset P.J., Gillette-Guyonnet S., Ritz P., Duveau F., Soto M.E., Provencher V. (2013). Cognitive frailty: Rational and definition from an (I.A.N.A./I.A.G.G.) international consensus group. J. Nutr. Health Aging.

[23] Hsu D.C., Mormino E.C., Schultz A.P., Amariglio R.E., Donovan N.J., Rentz D.M., Johnson K.A., Sperling R.A., Marshall G.A., Harvard Aging Brain S. (2016). Lower Late-Life Body-Mass Index is Associated with Higher Cortical Amyloid Burden in Clinically Normal Elderly. J. Alzheimers Dis..

[24] Sarma S., Sockalingam S., Dash S. (2021). Obesity as a multisystem disease: Trends in obesity rates and obesity-related complications. Diabetes Obes. Metab..

[25] Zhao Y., Wang H., Tang G., Wang L., Tian X., Li R. (2025). Risk factors for mild cognitive impairment in type 2 diabetes: A systematic review and meta-analysis. Front. Endocrinol..

[26] Cukierman T., Gerstein H.C., Williamson J.D. (2005). Cognitive decline and dementia in diabetes--systematic overview of prospective observational studies. Diabetologia.

[27] Huang P., Ren G., Wang Y., Liu Y., Zhang H., Fu S., Zhang Z., Guo L., Ma X. (2025). The Association Between the Atherogenic Index of Plasma and Cognitive Function: Evidence from the NHANES 2011–2014. Brain Behav..

[28] Zahodne L.B., Manly J.J., MacKay-Brandt A., Stern Y. (2013). Cognitive declines precede and predict functional declines in aging and Alzheimer’s disease. PLoS ONE.

[29] Zhao N., Liu C.C., Qiao W., Bu G. (2018). Apolipoprotein E, Receptors, and Modulation of Alzheimer’s Disease. Biol. Psychiatry.

[30] Yang L.G., March Z.M., Stephenson R.A., Narayan P.S. (2023). Apolipoprotein E in lipid metabolism and neurodegenerative disease. Trends Endocrinol. Metab..

[31] Kim Y.J., Seo S.W., Park S.B., Yang J.J., Lee J.S., Lee J., Jang Y.K., Kim S.T., Lee K.H., Lee J.M. (2017). Protective effects of APOE e2 against disease progression in subcortical vascular mild cognitive impairment patients: A three-year longitudinal study. Sci. Rep..

[32] Roda A.R., Montoliu-Gaya L., Villegas S. (2019). The Role of Apolipoprotein E Isoforms in Alzheimer’s Disease. J. Alzheimers Dis..

[33] Farrer L.A., Cupples L.A., Haines J.L., Hyman B., Kukull W.A., Mayeux R., Myers R.H., Pericak-Vance M.A., Risch N., van Duijn C.M. (1997). Effects of age, sex, and ethnicity on the association between apolipoprotein E genotype and Alzheimer disease. A meta-analysis. APOE and Alzheimer Disease Meta Analysis Consortium. JAMA.

[34] da Silva S.P., Lampraki C., Dos Santos Rego T., Ghisletta P., Kliegel M., Maurer J., Studer M., Gouveia E.R., de Maio Nascimento M., Ihle A. (2025). Can cognitive reserve offset APOE-related Alzheimer’s risk? A systematic review. Ageing Res. Rev..

[35] Dik M.G., Jonker C., Comijs H.C., Bouter L.M., Twisk J.W., van Kamp G.J., Deeg D.J. (2001). Memory complaints and APOE-epsilon4 accelerate cognitive decline in cognitively normal elderly. Neurology.

[36] Striepens N., Scheef L., Wind A., Meiberth D., Popp J., Spottke A., Kolsch H., Wagner M., Jessen F. (2011). Interaction effects of subjective memory impairment and ApoE4 genotype on episodic memory and hippocampal volume. Psychol. Med..

[37] Backman K., Joas E., Waern M., Ostling S., Guo X., Blennow K., Skoog I., Gustafson D.R. (2015). 37 Years of Body Mass Index and Dementia: Effect Modification by the APOE Genotype: Observations from the Prospective Population Study of Women in Gothenburg, Sweden. J. Alzheimers Dis..

[38] Shinohara M., Gheni G., Hitomi J., Bu G., Sato N. (2023). APOE genotypes modify the obesity paradox in dementia. J. Neurol. Neurosurg. Psychiatry.

[39] Vidoni E.D., Townley R.A., Honea R.A., Burns J.M., Alzheimer’s Disease Neuroimaging I. (2011). Alzheimer disease biomarkers are associated with body mass index. Neurology.

[40] Zhai W., Zhang G., Wei C., Zhao M., Sun L. (2025). The obesity paradox in cognitive decline: Impact of BMI dynamics and APOE genotypes across various cognitive status. Diabetes Obes. Metab..

[41] Chen H., Zhou T., Guo J., Ji J.S., Huang L., Xu W., Zuo G., Lv X., Zheng Y., Hofman A. (2022). Association of Long-Term Body Weight Variability with Dementia: A Prospective Study. J. Gerontol. A Biol. Sci. Med. Sci..

[42] Christensen D.Z., Schneider-Axmann T., Lucassen P.J., Bayer T.A., Wirths O. (2010). Accumulation of intraneuronal Abeta correlates with ApoE4 genotype. Acta Neuropathol..

[43] Blautzik J., Kotz S., Brendel M., Sauerbeck J., Vettermann F., Winter Y., Bartenstein P., Ishii K., Rominger A., Alzheimer’s Disease Neuroimaging Initiative (2018). Relationship Between Body Mass Index, ApoE4 Status, and PET-Based Amyloid and Neurodegeneration Markers in Amyloid-Positive Subjects with Normal Cognition or Mild Cognitive Impairment. J. Alzheimers Dis..

[44] Bobo R.H., Riahi S., Ojha V.P., Yarahmadian S. (2025). An Analysis of the Relationship Between the APOE4 Allele Count, Age of Onset, and Cognitive Impairment Prevalence in the NACC Database: Evaluating the Nigerian Paradox. J. Dement. Alzheimer’s Dis..

